# Miscellany

**Published:** 1910-05

**Authors:** 


### MISCELLANY.

---

Medical Interne.—Government Hospital for the Insane, June 15, 1910.—The United States Civil Service commission announces an examination on June 15, 1910, at the usual places, to secure eligibles from which to make certification to fill at least two vacancies in the position of medical interne (male), Government Hospital for the Insane, Washington, D. C., at $600 per annum each, with maintenance, and vacancies requiring similar qualifications as they may occur in that hospital, unless it shall be decided in the interests of the service to fill either or both of the vacancies by reinstatement, transfer, or promotion. From the grade of medical interne the hospital makes promotions to the higher positions in the medical staff as vacancies occur.

Both men and women will be admitted to this examination, although there are no vacancies for women at present. Applicants must be unmarried. Age limit, 20 years or over on the date of the examination.

This examination is open to all citizens of the United States who comply with the requirements.

Applicants should at once apply either to the United States Civil Service Commission, Washington, D. C., or to the secretary of the board of examiners at any place mentioned in the list printed heretofore, for application Form 1312. No application will be accepted unless properly executed and filed with the Commission at Washington. In applying for this examination the exact title as given at the head of this announcement should be used in the application.

As examination papers are shipped direct from the Commission to the places of examination, it is necessary that applications be received in ample time to arrange for the examination desired at the place indicated by the applicant. The Commission will therefore arrange to examine any applicant whose application is received in time to permit the shipment of the necessary papers.

Issued April 8, 1910.

Army Medical Corps Examination.—The Surgeon General of the Army announces that preliminary examination of applicants for appointment as First Lieutenants in the Army Medical Corps, will be held on July 18, 1910, at various army posts throughout the country.

Full information concerning the examination can be procured upon application to the "Surgeon General. U. S. Army, Washington, D. C." The essential requirements to securing an invitation are that the applicant shall be a citizen of the United States, shall be between 22 and 30 years of age, a graduate of a medical school legally authorised to confer the degree of doctor of medicine, shall be of good moral character and habits, and shall have had at least one year's hospital training, or its equivalent in practice. The examination will be held concurrently throughout the country at points where boards can be convened. Due consideration will be given to localities from which applications are received, in order to lessen the traveling expenses as much as possible.

The examination in subjects of general education (mathematics, geography, history, general literature and Latin) may be omitted in the cast of applicants holding diplomas from reputable literary or scientific colleges, normal schools or high schools, or graduates of medical schools which require an entrance examination satisfactory to the faculty of the Army Medical School.

In order to perfect all necessary arrangements for the examination, applications must be complete and in possession of The Adjutant General on or before June 27, 1910. Early attention is therefore enjoined upon all intending applicants. There are at present 123 vacancies in the Medical Corps of the Army.

---

Our advertising forms present the advertisement of the Radu Surgical Instrument Co., which should be of interest to most physicians. This company is recently organised but in its equipment and in the excellency of its instruments and for skilled workmanship, it stands well in the lead. In the general diagnostic case a cut of which is given, attention is especially called to the superiority of the "Cold lamps" in the electrically lighted instruments. All instruments in the case are guaranteed to be mechanically true and made of the best German silver. None but silver solder is used in the joints. In addition to the general diagnostic case The Radu Company are makers of a large line of special surgical instruments and diagnostic apparatus, such as high frequency and *x*-ray machines. Send for catalogue.

